# Advanced phosphocreatine-grafted chitosan hydrogel promote wound healing by macrophage modulation

**DOI:** 10.3389/fbioe.2023.1199939

**Published:** 2023-05-12

**Authors:** Weibei Sheng, Haotian Qin, Tiehua Wang, Jin Zhao, Chongzhou Fang, Peng Zhang, Peng Liu, Anjaneyulu Udduttula, Hui Zeng, Yingqi Chen

**Affiliations:** ^1^ Department of Bone & Joint Surgery, National & Local Joint Engineering Research Center of Orthopaedic Biomaterials, Peking University Shenzhen Hospital, Shenzhen, China; ^2^ Department of Emergency, Shenzhen People’s Hospital, Shenzhen, China; ^3^ Central Laboratory, Peking University Shenzhen Hospital, Shenzhen, China; ^4^ Centre of Biomaterials, Cellular & Molecular Theranostics (CBCMT), Vellore Institute of Technology (VIT), Vellore, India

**Keywords:** phosphocreatine-grafted chitosan hydrogel, wound healing, macrophage modulation, NF-κB signaling pathway, tissue engineering

## Abstract

**Background:** The repair of wounds usually caused by trauma or other chronic diseases remained challenging in clinics due to the potential risk of inflammation and inadequate tissue regenerative properties. Among them, the behaviour of immune cells, such as macrophages, is critical in tissue repair.

**Materials and methods:** In this study, a water-soluble phosphocreatine-grafted methacryloyl chitosan (CSMP) was synthesized with a one-step lyophilization method, followed by the fabrication of CSMP hydrogel with a photocrosslinked method. The microstructure, water absorption and mechanical properties for the hydrogels were investigated. Then, the macrophages were co-cultured with hydrogels and the pro-inflammatory factors and polarization markers for these macrophages were detected through real-time quantitative polymerase chain reaction (RT-qPCR), Western blot (WB), and flow cytometry methods. Finally, the CSMP hydrogel was implanted in a wound defect area in mice to test its ability to promote wound healing.

**Results:** The lyophilized CSMP hydrogel had a porous structure with pores ranging in size from 200 to 400 μm, which was larger than the CSM hydrogel’s. The lyophilized CSMP hydrogel possessed a higher water absorption rate compared with the CSM hydrogel. The compressive stress and modulus of these hydrogels were increased in the initial 7 days immersion and then gradually decreased during the *in vitro* immersion in PBS solution up to 21 days; the CSMP hydrogel showed a higher value in these parameters versus the CSM hydrogel. The CSMP hydrogel inhibited the expression of inflammatory factors such as interleukin-1β (IL-1β), IL-6, IL-12, and tumor necrosis factor-α (TNF-α) in an *in vitro* study cocultured with pro-inflammatory factors in pre-treated bone marrow-derived macrophages (BMM). The mRNA sequencing results showed that the CSMP hydrogel might inhibit the macrophages’ M1 type polarization through the NF-κB signaling pathway. Furthermore, when compared to the control group, the CSMP hydrogel promoted more skin area repair in the mouse wound defect area, and inflammatory factors such as IL-1β, IL-6, and TNF-α were lower in the repaired tissue for the CSMP group.

**Conclusion:** This phosphate-grafted chitosan hydrogel showed great promise for wound healing through regulating the macrophage’s phenotype via the NF-κB signaling pathway.

## 1 Introduction

Wound defects, usually caused by chronic disease, age-related disease, or others, are one of the most common diseases seen in clinics, and the treatment of this disease is difficult. The current treatment methods in clinics mainly include local drug treatment, systemic administration, and surgical treatment. The frequently used drugs contain growth factors, anti-bacterial drugs, blood supplying drugs, or others. However, these treatment methods are expensive, require multiple and frequent drug administration, and have other side effects. In the last decade, wound dressings made from hydrogels, alginate, or other natural components showed great promise for wound healing, and some of them have been used in clinics (Rezvani Ghomi et al., 2019).

In recent, various novel biomaterials have been developed to deal with the wound healing, including natural polysaccharide-based materials (Chen et al., 2021), such as hyaluronic acid based biomaterials (Zhang et al., 2022), chitosan-based biocomposites (Shu et al., 2023), polysaccharide-based hydrogels (Abazari et al., 2022), and other synthetic polymeric scaffolds, such as polyphosphate (Müller et al., 2023), electrospun poly(L-lactic-co-caprolactone) (PCL) nanofilm (Wang et al., 2022), PCL and collagen mixed electrospun scaffold (Ghosh et al., 2022), 3D printed scaffold (Hu et al., 2021), and others (Suamte et al., 2023). These attempts showed attractive results for wound healing in terms of anti-bacterial properties and defect healing properties, but some of them still had insufficiencies, such as unsatisfied wound repair efficacy, complicated fabrication methods or equipment, acidic degradation products (usually stimulating the inflammatory response), or others for clinical use or manufacturing production. As a result, it is critical to continue developing novel biomaterials that are primarily composed of natural or human body-derived components and have excellent tissue repair-promoting ability, biocompatibility, biodegradability, and other properties.

To this end, hydrogels, especially those made from natural polysaccharides, showed great potential for wound healing, as the hydrogels possess a high water content and a micro- and/or nanobiomimetic three-dimensional structure that simulates the natural extracellular matrix (ECM), benefiting the transport of nutrients and metabolic wastes, and cells’ attachment and proliferation (Hamedi et al., 2018; Tian et al., 2021). Besides, the moist environment of dressed hydrogels would also physically block the exterior bacteria from reaching the wound site to avoid further infection. In the natural polysaccharide-based hydrogels, chitosan is a kind of natural product that is extracted from mussel shells and which possesses attractive attributes for wound healing, including anti-bacterial properties, biocompatibility, biodegradability, hemostasis, and even immunomodulatory properties (Yeh et al., 2016); therefore chitosan and its derivate are frequently used as a kind of hemostatic or wound dressing biomaterial (Alves and Mano, 2008; Ribeiro et al., 2009; Cao et al., 2020). However, the chitosan suffers from low water solubility in pure water, which remains a challenge for the fabrication of hydrogels or other biomaterials. Grafting some micromolecules or functional groups into chitosan, such as carboxymethyl (Kłosiński et al., 2023), methacrylic acid, organic phosphates (Chen Y. et al., 2022), or others (Muñana-González et al., 2023) would improve its water solubility. Among them, the methacrylic acid would be regarded as one of the better choices, as the methacryloyl chitosan could be dissolved in pure water and further photocrosslinked with a low-toxic initiator to form the hydrogel. Besides, organic phosphates would also be a good option, as the element phosphate, an essential composition in the human body, participates in various metabolic reactions and wound healing.

In the tissue regeneration process, the immune response and/or immune modulation play an essential role in tissue regeneration. Immune cells’ behavior, e.g., phenotypic change, determines the tissue regeneration capacity. The macrophage is a type of commonly occurring immune cell that is found in many organs or tissues (Murray and Wynn, 2011). Usually, macrophages are divided into two different cell subtypes, M1 and M2, that play important roles in different stages of tissue repair after injury. M1 macrophage cells are pro-inflammatory and recruit immune cells to damaged tissue, and this type of macrophage is usually induced to express a large number of pro-inflammatory factors, such as IL-12 and TNF-α, by Toll-like ligands such as lipopolysaccharide (LPS) or interferon gamma (IFNγ) (Murray, 2017). M2-type macrophages are beneficial for eliminating the inflammatory phase and promoting damaged tissue repair (Murray, 2017). M2-type macrophages are beneficial for eliminating the inflammatory phase and promoting damaged tissue repair (Mahdavian Delavary et al., 2011), while this type of macrophage is typically induced by IL-4 or IL-13 and expresses less pro-inflammatory factors but more anti-inflammatory factors such as IL-10 (Murray, 2017). Once the tissue was damaged, in response to chemotactic signals, the monocytes would migrate into the damaged tissue and subsequently differentiate into M1 macrophages; these macrophages would stimulate the inflammatory response to resist the foreign damage in the initial stage. During the tissue repair process, macrophages would swallow the apoptotic cells. Meanwhile, as tissue repair progresses, M2 macrophages will predominate in promoting tissue repair. Therefore, regulating the macrophage phenotype, especially the balance and ratio of M1 and M2 macrophages, plays a key role in the healing and remodeling of damaged tissues and can even accelerate tissue repair (Mahdavian Delavary et al., 2011). So far, although numerous different kinds of biomaterials have been developed to modulate the macrophage phenotype to promote tissue repair (Han et al., 2019; Xie et al., 2019), to our best knowledge, few studies have concentrated on developing chitosan-based hydrogels without the addition of other exogenous bioactive components for macrophage modulation to promote wound healing.

Herein, natural polysaccharide chitosan was successively grafted with methacrylic acid and phosphocreatine by the one-step lyophilization method to synthesize the photocrosslinkable phosphate-functionalized methacryloyl chitosan (CSMP), and then the CSMP hydrogel was fabricated with the addition of cross-linking agents and an ultraviolet (UV) initiator under UV irritation. This fabricated hydrogel was aimed at promoting the wound healing through macrophage modulation. To the best of our knowledge, this was the first-time that phosphocreatine was grafted in a chitosan-based hydrogel to promote wound healing via macrophage modulation. Phosphocreatine is a kind of energy-rich phosphate that participates in almost all the energy metabolism in the human body (Walter et al., 1997). It widely exists in muscle and other excitable tissues. Reported studies confirmed that phosphocreatine would modulate the phenotype of macrophages (Weischenfeldt and Porse, 2008), indicating a high potential for macrophage modulation in wound healing. SEM was used to examine the surface morphology of lyophilized hydrogels, and the variations in compressive mechanical properties for this CSMP hydrogel *in vitro* immersion in PBS solution for up to 21 days were also investigated in comparison to CSM hydrogel. The immune modulation ability of this CSMP hydrogel, in terms of promoting macrophage polarization and expression of inflammatory factors, was studied *in vitro*, and the mechanism was also explored with the mRNA sequencing method. Further, the hydrogel was implanted in the skin defect area in mice to illustrate the skin’s regenerative capacity.

## 2 Materials and methods

### 2.1 Synthesis of phosphocreatine-modified chitosan hydrogel

In 300 mL of 1% acetic acid water solution, 3 g chitosan was dissolved. Then, 1.1 mL of methacrylic anhydride was added to this chitosan acetic acid solution, followed by the reaction at room temperature for 24 h. Meanwhile, 1.17 g phosphocreatine (PS) was dissolved in 150 mL of 0.5M 2-(N-morpholino) ethanesulfonic acid monohydrate (MES) solution, and then 3.5 g N-(3-dimethylamino propyl)-N′-ethylcarbodiimide hydrochloride (EDC) and 1.17 g of N-hydroxysuccinimide (NHS) were dissolved in this PS solution. After that, the methacrylic anhydride reacted chitosan solution was dropwise added to the PS solution, and after being stirred at room temperature for 24 h, the reacted solution was dialyzed in DI water for 1 week (the water was changed twice per day). Then, the dialyzed solution was lyophilized to obtain the white flocculent CSMP. For the control, the methacrylic anhydride reacted chitosan solution was directly dialyzed and lyophilized to obtain the flocculent methacryloyl chitosan (CSM). For the hydrogel preparation, the CSM and CSMP were respectively dissolved in DI water at a concentration of 20 mg/mL, and then 10 mg/mL acrylamide, 0.2 mg/mL BIS, and 1 mg/mL I2959 were added and dissolved into this CSMP solution. The mixed pre-gel solution was placed in a syringe, which was exposed to UV irradiation for 30 min to form the hydrogel. The hydrogel was cut into a cylindrical shape and stored in a refrigerator at 4°C for further experiments.

### 2.2 Characterization

The cylindrical hydrogel was lyophilized, followed by the gold spray. Then, the surface morphology of lyophilized hydrogel was observed with a scanning electron microscope (SEM, JEOL JSM-7401F, Tokyo, Japan).

### 2.3 Water uptake test

The as-fabricated cylindrical hydrogels (8 mm in diameter and 8 mm in height) were lyophilized materials, and then the lyophilized were weighted as g_0_. After that, the lyophilized hydrogels were immersed in DI water for 2 hours, and then the samples were taken out, and after wiping the water, the hydrogels were weighted as g_1_, followed by the calculation of the water uptake ratio with g_1_/g_0_ × 100%.

### 2.4 Compressive properties of hydrogel in the *in vitro* degradation process

The hydrogel was cut into a cylindrical shape (a diameter of 8 mm and a height of 10 mm) and then immersed in PBS solution in a 37°C incubator for 7, 14, and 21 days. At the set time point, the PBS solution was removed, and the compression properties were measured with a static and dynamic materials testing machine (Instron-E3000, Norwood, MA, US) with a load of 250 N and a compression speed of 5 mm/min. The stress-strain curves were obtained, and the compressive stress and compressive modulus were calculated from these curves.

### 2.5 Extraction and identification of bone marrow-derived macrophages (BMMs)

BMMs were extracted according to the method provided by (Weischenfeldt and Porse, 2008). Briefly, 8-week-old C57 female mice were sacrificed by CO_2_ anesthesia, and then the femurs were obtained. After cutting both ends of the femurs, the bone marrow was flushed with a syringe filled with 1640 medium (SH30809.01, Hyclone, US). The cell suspension was then filtered through a 70 μm cell strainer and centrifuged at 500 *g* for 8 min. After that, the precipitate was resuspended in 1640 medium containing 20% L929 culture medium and 10% FBS, and the cells were seeded in a 24-well plate with 700 μL medium, including 2 × 10^6^ cells per well. The medium was refreshed on the third, fifth, and sixth days. For the stimulation of M1 or M2 macrophages, 10 ng/mL LPS (Sigma, L2630) and 10 ng/mL IFNγ (PeproTech, 400-20-20UG) or 10 ng/mL IL-4 (PeproTech, 400-04-20UG) were respectively added to 7-day cultured macrophages, and after 24 h of culture, the M1 or M2 macrophages were obtained. F4/80, a highly glycosylated G protein-coupled receptor, and an established marker are usually used to identify the mouse macrophages (McKnight et al., 1996). For the F4/80 staining, the cells were fixed with 4% paraformaldehyde (China, Solarbio, P1110) for 20 min, and after three washes with PBS, the cells were blocked with QuickBlockTM blocking solution (China, Beyotime, P0265) for 1 h. Then, the cells were incubated with F4/80 primary antibody (China, ABclonal, A18637) overnight, and after washed with PBS the cells were incubated with specific secondary antibody. After being washed with PBS, the cells were stained with an anti-fluorescence quencher containing DAPI (China, Beyotime, P0131). Finally, the cells were observed under a fluorescent microscope.

### 2.6 RT-qPCR

For the RT-qPCR assay, 5 × 10^5^ BMMs were first co-cultured with CSM or CSMP samples for 12 h, and then the cells were cultured with medium containing 10 ng/mL LPS and 10 ng/mL IFNγ for 12 h. The cells were then digested with 0.25% trypsin, and the total RNA for each sample was extracted according to the protocol of the RNA extraction kit (DP419, TIANGEN BIOTECH, China). The extracted RNA was then reverse-transcribed to cDNA with the RT reagent kit (AG11706, ACCURATE BIOLOGY, China), and the cDNA was used to assay by real time fluorescence quantitative PCR instrument (LightCycler 96, Roche, China). IL-1β, IL-6, TNF-α, CC chemokine ligand 2 (CCL2), inducible nitric oxide synthase (iNOS), IL-12β, and CD86 mRNA levels were measured and normalized to the internal control GAPDH. Table 1 contains a list of gene primer sequences. The number of parallel samples for statistical purposes was no less than three per assay, and each assay was performed at least twice.

**TABLE 1:** **Primer sequences of genes**.

Real-time quantitative PCR primer sequence	
Gene	Sequence (5’- 3’ on minus strand)
*GAPDH*	Fwd: TGTGTCCGTCGTGGATCTGA
	Rev: TTGCTGTTGAAGTCGCAGGAG
*IL-1* β	Fwd: TCCAGGATGAGGACATGAGCAC
	Rev: GAACGTCACACACCAGCAGGTTA
*IL-6*	Fwd: CCACTTCACAAGTCGGAGGCTTA
	Rev: CCAGTTTGGTAGCATCCATCATTTC
*IL-12* β	Fwd: TCTTTGTTCGAATCCAGCGCA
	Rev: AGACATTCCCGCCTTTGCATT
*TNF-α*	Fwd: ACTCCAGGCGGTGCCTATGT
	Rev: GTGAGGGTCTGGGCCATAGAA
*CCL2*	Fwd: AGCAGCAGGTGTCCCAAAGA
	Rev: GTGCTGAAGACCTTAGGGCAGA
*iNOS*	Fwd: CAAGCTGAACTTGAGCGAGGA
	Rev: CAAGCTGAACTTGAGCGAGGA
*CD86*	Fwd: AGAAGCCGAATCAGCCTAGCA
	Rev: CTGCCTTCACTCTGCATTTGGT
*Lipg*	Fwd: TACTCTCCCTGTCGCTAGGTG
	Rev: TCTACAGTCCCCTCTCTATGGC
*MMP25*	Fwd: ATTGCAGCTCCTCAAACCAGA
	Rev: TCCTGGTCCTGTTTAACGCAT
*TLR9*	Fwd: TCTTCATGTCTGGTTCCCGAGT
	Rev: TACAGCCAACCTTTGCTCCG
*Dnase1l3*	Fwd: ATCCTTGCCCTCCATGACAC
	Rev: CAACAGTATAAGGTCACAGCGTT
*Ikbkb*	Fwd: CCGGATCCAGCTCTCTTAGACA
	Rev: ACTACAGCATAGTCAAAGGTCGTT
*Nfkb1*	Fwd: TGCCAAAGAAGGACACGACA
	Rev: TGAGCATTGACTTCTGCCCC
*Nfkb2*	Fwd: CTAGTCACCAAGCTCCATGCT
	Rev: GGAGGCGAGTAAGAGTTGGG

### 2.7 mRNA sequencing

For the mRNA sequencing assay, 5 × 10^5^ BMMs were first co-cultured with the hydrogel sample for 12 h, and then the cells were cultured with medium containing 10 ng/mL LPS and 10 ng/mL IFNγ for 12 h mRNA sequencing was performed by Bgi Genomics corporation according to the protocol. The differential expression genes between different groups were screened out, and the enriched signaling pathways of differential expression genes were analyzed by KEGG pathway enrichment.

### 2.8 Western blot

For Western blot (WB) tests, 5 × 10^5^ BMMs treated with LPS and IFNγ or IL-4 were cultured with CSMP for 24 h. The total protein was then harvested in a RIPA lysis solution (China, Beyotime, P0013B). The obtained proteins were separated by sodium dodecyl sulfate-polyacrylamide gel electrophoresis (SDS-PAGE) and then transferred onto polyvinylidene fluoride (PVDF) membranes using a trans-buffer at 300 A for 60 min. After blocking with 5% skim milk for 1 h, the primary antibodies were incubated overnight and then washed with TBST three times, followed by incubation with specific secondary antibody (ab205718, Abcam, USA). After washing TBST for 30 min, the membranes with the proteins were visualized with the BeyoECL plus kit (WBKLS0500, Milipore, USA). For WB testing, incubated primary antibodies included IL-1β (12242S, CST, USA), Arginase1 (Arg1) (D4E3M, CST, USA).

### 2.9 Flow cytometry

For flow cytometry tests, 5 × 10^5^ BMMs treated with IL-4 were cultured with CSMP for 24 h with the same cell culture method presented above, and the cells were then digested with trypsin. The cells were blocked with purified rat Anti-Mouse CD16/CD32 (553142, BD Pharmingen™, USA) for 20 min. The cells were then incubated with BV510 Rat Anti-Mouse F4/80 (74328, BD Pharmingen, USA), followed by fixation and permeation. The cells were then incubated with Arg1 Monoclonal Antibody, PE-Cyanine7 (25-3697-82, eBioscience, USA) and iNOS Monoclonal Antibody, PE (12-5920-82, eBioscience, USA), and the cells were measured with flow cytometry according to the protocol.

### 2.10 Surgical procedures and hydrogel implantation

Female BALB/c mice (8-week-old) were purchased from the Guangdong Medical Laboratory Animal Center (Guangzhou, China) and randomly divided into two groups (*n* = 4 for each group). All surgical procedures were performed following the instructions approved by the Institutional Animal Care and Use Committee (IACUC) of Peking University Shenzhen Hospital, and the IACUC number was 2022-164. The animals were anesthetized with isoflurane using a gas anesthesia machine. After each mouse’s back was shaved, the skin was fully exposed. The defect (8 mm in diameter) was created with scissors, and a defect was created in each mouse. The disc hydrogel samples (8 mm in diameter, 1 mm in height) were implanted in the defect area. The gross wounds were imaged at 0, 5, and 10 days of implantation, and the wound contraction was calculated from these images using the ImageJ software (National Institutes of Health, Bethesda, USA). After 10 days of implantation, the mice were sacrificed with excessive anesthesia, and the wound defect area was obtained for further investigations.

### 2.11 Histological assessment and immunohistochemical staining

The harvested skins were fixed in 10% formalin for 1 week; subsequently, the tissues were embedded in paraffin, and the cross sections of skin at the central area of the defect were cut at 5 μm for hematoxylin and eosin (H&E) and immunohistochemical staining for CD31 (1:100, A0378, Abcalonal, China).

### 2.12 RT-qPCR, flow cytometry and Western blot

Skin samples, including the wound and a 4-mm margin of surrounding skin, were harvested at day 10 post-surgery, snap-frozen in liquid nitrogen, and stored at −80 °C for further RT-qPCR studies. The subsequent operation steps are the same as step 2.6. The mRNA levels of the IL-1β, IL-6, and TNF-α were detected.

The harvested skins were digested in PBS containing 1 mg/mL collagenase, 60 U/mL DNase and 2% FBS for 1 h at 37°C to obtain the cells. The digested cells were blocked with purified rat Anti-Mouse CD16/CD32 (553142, BD Pharmingen™, USA) for 20 min. Then, the cells were incubated with 7-AAD (ST515, Beyotime Biotechnology, China), APC-Cy™7 Rat Anti-Mouse CD45 (557659, BD Pharmingen, USA), Alexa Fluor® 488 Rat Anti-mouse CD11b (557672, BD Pharmingen, USA), and BV510 Rat Anti-Mouse F4/80 (743280, BD Pharmingen, USA), followed by fixation and permeation. The cells were then incubated with Arg1 Monoclonal Antibody, PE-Cyanine7 (25-3697-82, eBioscience, USA) and iNOS Monoclonal Antibody, PE (12-5920-82, eBioscience, USA), and the cells were measured with flow cytometry according to the protocol.

Total protein was extracted from the wound samples with RIPA (Beyotime, China) for at least 30 min. Total protein samples per group were heated with SDS-PAGE sample buffer and separated on a 12% SDS-PAGE gel. Proteins were then transferred to a PVDF membrane in a transfer buffer and blocked for 1 h in 5% skim milk in TBST. The membranes containing the transferred proteins were incubated overnight at 4°C with primary antibodies against Arg1 and then washed with TBST three times, followed by incubation with specific secondary antibody (ab205718, Abcam, USA). After washing TBST for 30 min, the membranes with the proteins were visualized with the BeyoECL plus kit (WBKLS0500, Milipore, USA).

## 3 Results

### 3.1 Characterization of phosphocreatine-modified chitosan hydrogels

**FIGURE 1:**
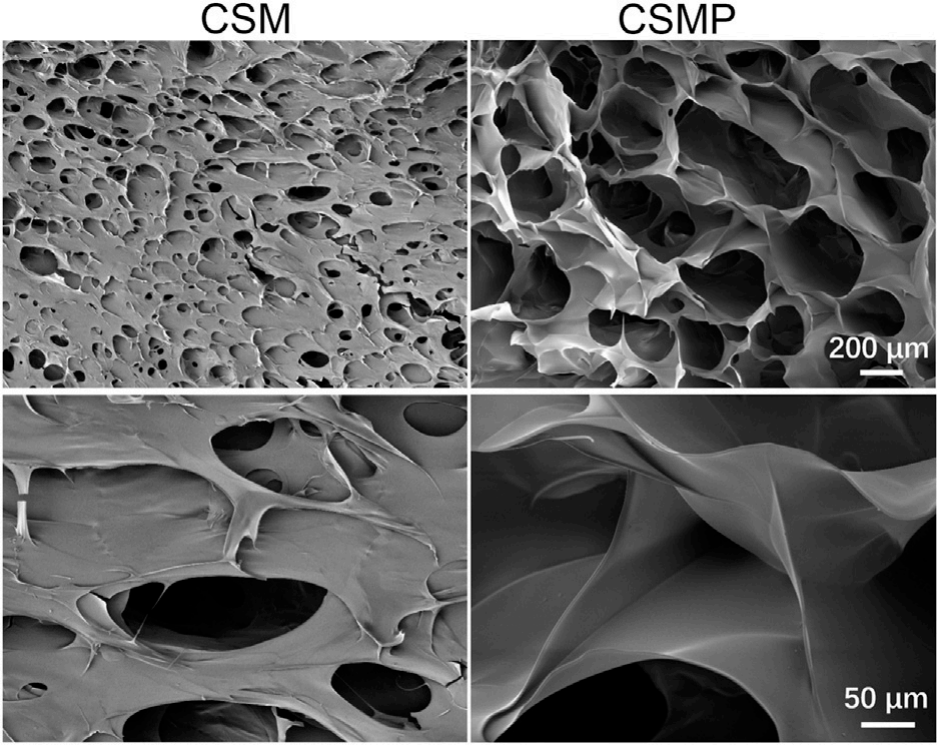
Surface SEM images for the lyophilized CSM and CSMP hydrogels.

The surface SEM images for the lyophilized hydrogels were showed in Figure 1. As shown, these two lyophilized hydrogels presented porous structures, but the pore size for CSMP (ca. 200 μm) was larger and deeper than that of the CSM hydrogel (pore size was ca. 100 μm).

### 3.2 Water absorption properties of lyophilized hydrogels

**FIGURE 2:**
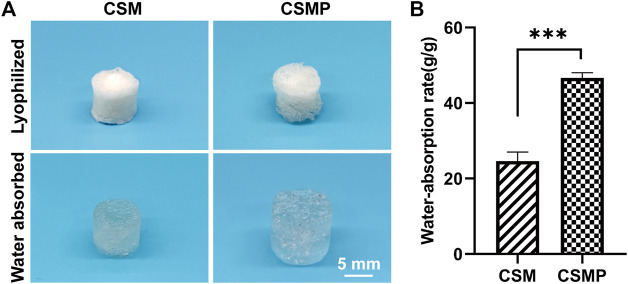
Water absorption of the lyophilized CSM and CSMP hydrogels. **(A)** Macroscopic images for the hydrogels before and after water absorption test, **(B)** the statistical water absorption rate.

The water absorption of lyophilized hydrogels was performed, and the result was shown in Figure 2. As shown in macroscopical images in Figure 2A, CSMP lyophilized hydrogel absorbed more water than that of CSM hydrogel, and the statistical water-absorption result in Figure 2B confirmed that the water-absorption amount for lyophilized CSMP hydrogel was almost two times larger than that of the CSM lyophilized hydrogel.

### 3.3 Compressive properties of hydrogels during the *in vitro* degradation process

**FIGURE 3:**
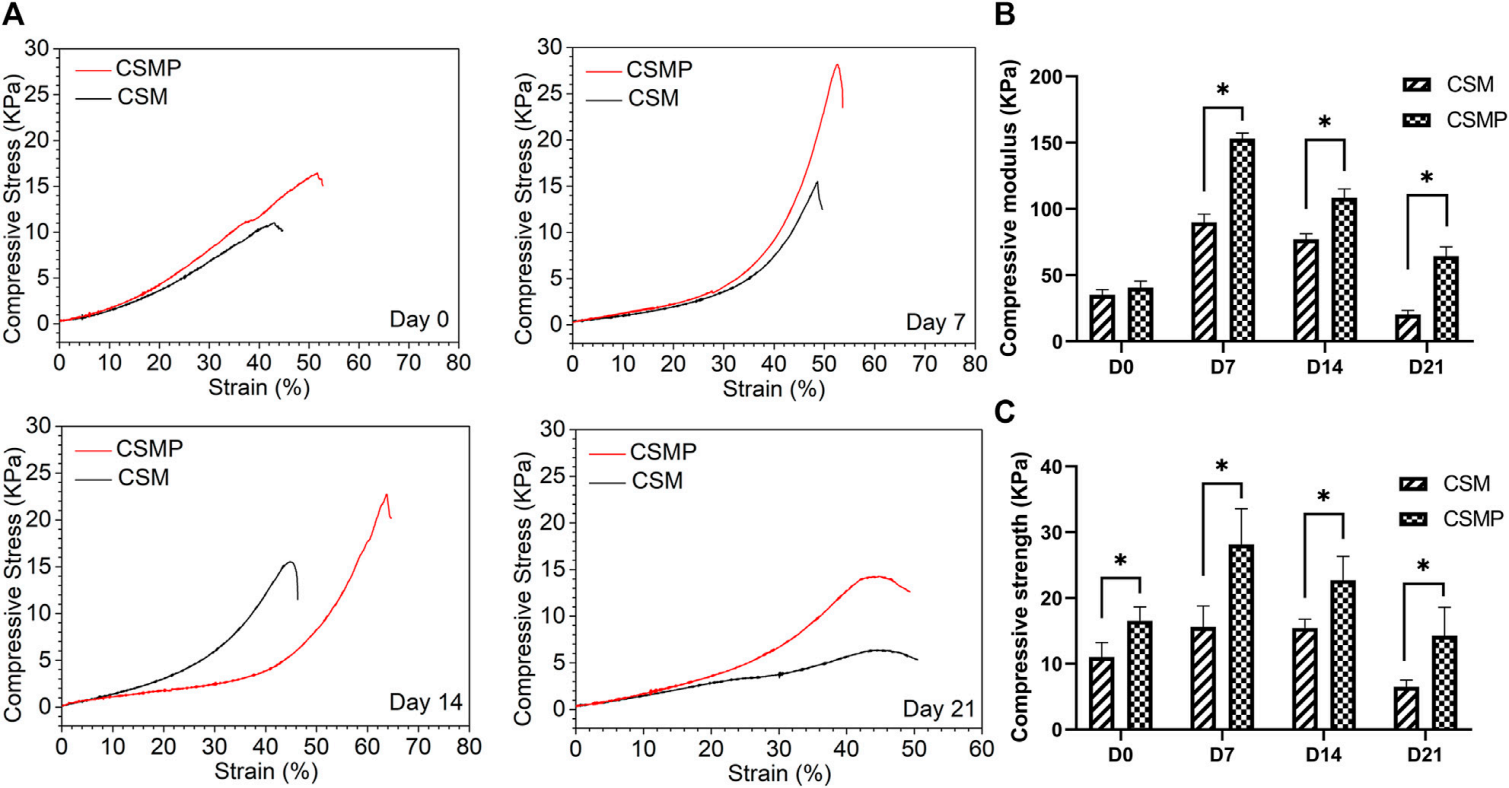
Compressive properties of hydrogels during the *in vitro* immersion in PBS solution at 37°C up to 21 days. **(A)** The representative stress-strain curves for the hydrogels after immersion in PBS for 0, 7, 14, and 21 days. **(B)** and **(C)** The statistical analysis results for compressive modulus and strength of hydrogels after immersion in PBS for 0, 7, 14, and 21 days.

The compressive properties of hydrogels statically immersed in PBS for 0, 7, 14, and 21 days were evaluated, and the results were shown in Figure 3. The CSMP hydrogel had a higher compressive strength than the CSM hydrogel at each detected time point, according to representative strain vs. stress curves in Figure 3A. The statistical compressive modulus and strength results in Figures 3B, C confirmed the result presented in Figure 3A. At each time point, the compressive modulus and strength of the CSMP hydrogel were greater than those of the CSM hydrogel (except at the day 0 time point for the compressive modulus). Meanwhile, the compression properties for these two hydrogels showed the same tendency: they increased at the initial 7-day immersion and then gradually decreased during the *in vitro* immersion process.

### 3.4 BMMs’ identification and culture

F4/80 is the surface marker for BMMs, and the F4/80 staining was performed to extract BMMs. The result was displayed in Supplementary Figure S1. From Supplementary Figure S1A, the extracted cells were stained with the F4/80 marker, indicating these cells are BMMs. After being treated with LPS and IFNγ or IL-4 for 24 h, the cell morphology was different with the extracted BMMs, as shown in Supplementary Figures S1D–F.

### 3.5 BMM cells’ behavior when cultured with hydrogels

**FIGURE 4:**
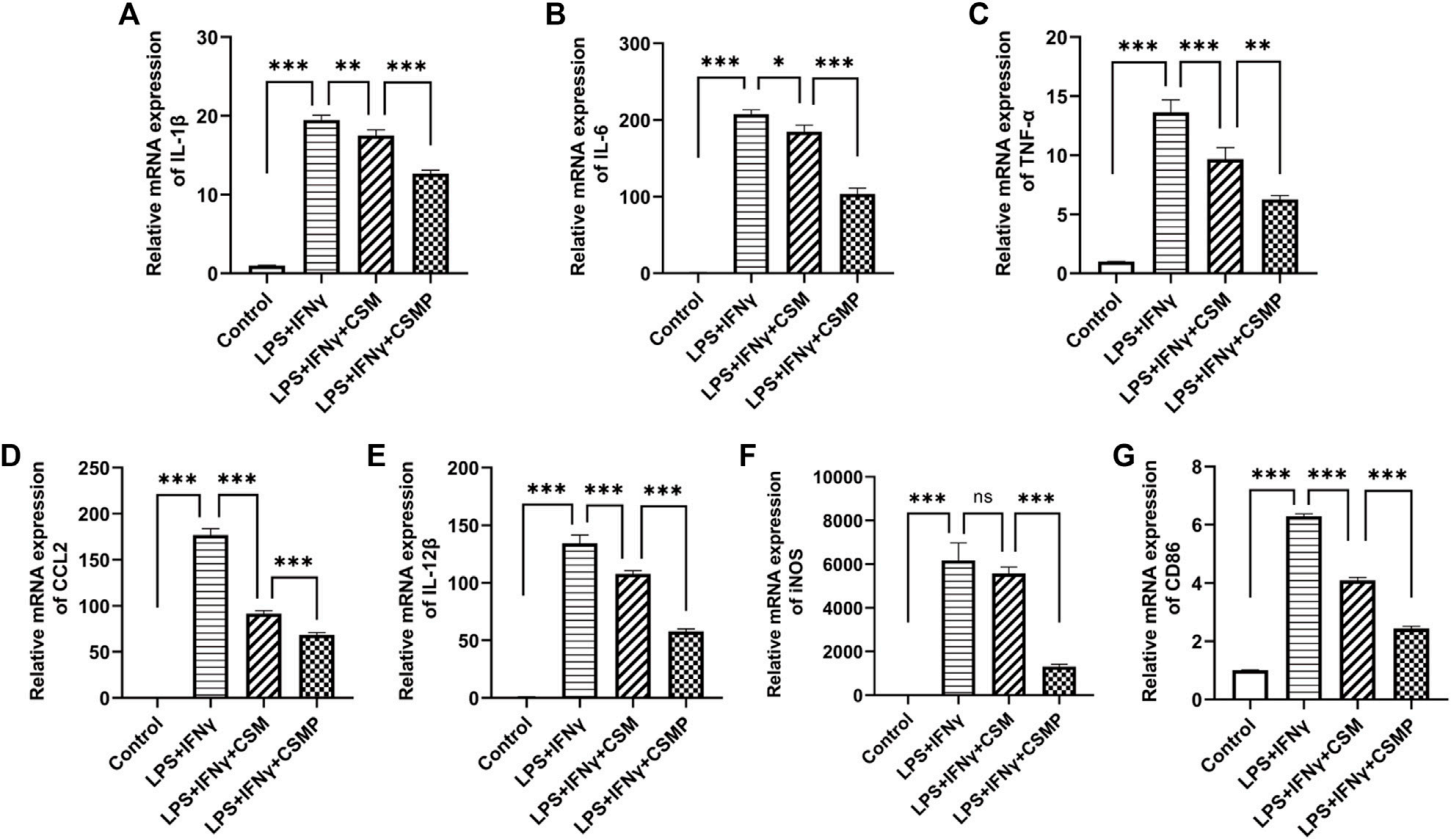
The RT-qPCR results for the pro-inflammatory factors stimulated BMMs after cocultured with CSMP or CSM hydrogel. **(A–E)** mRNA expression for pro-inflammatory related genes, IL-1β, IL-6, IL-12β, TNF-α, CCL2. **(F,G)** mRNA expression level for iNOS and CD86 genes, which are the characteristic genes for M1 type macrophages. (*n* = 3, *p < 0.05, **p < 0.01, ***p < 0.001).

**FIGURE 5:**
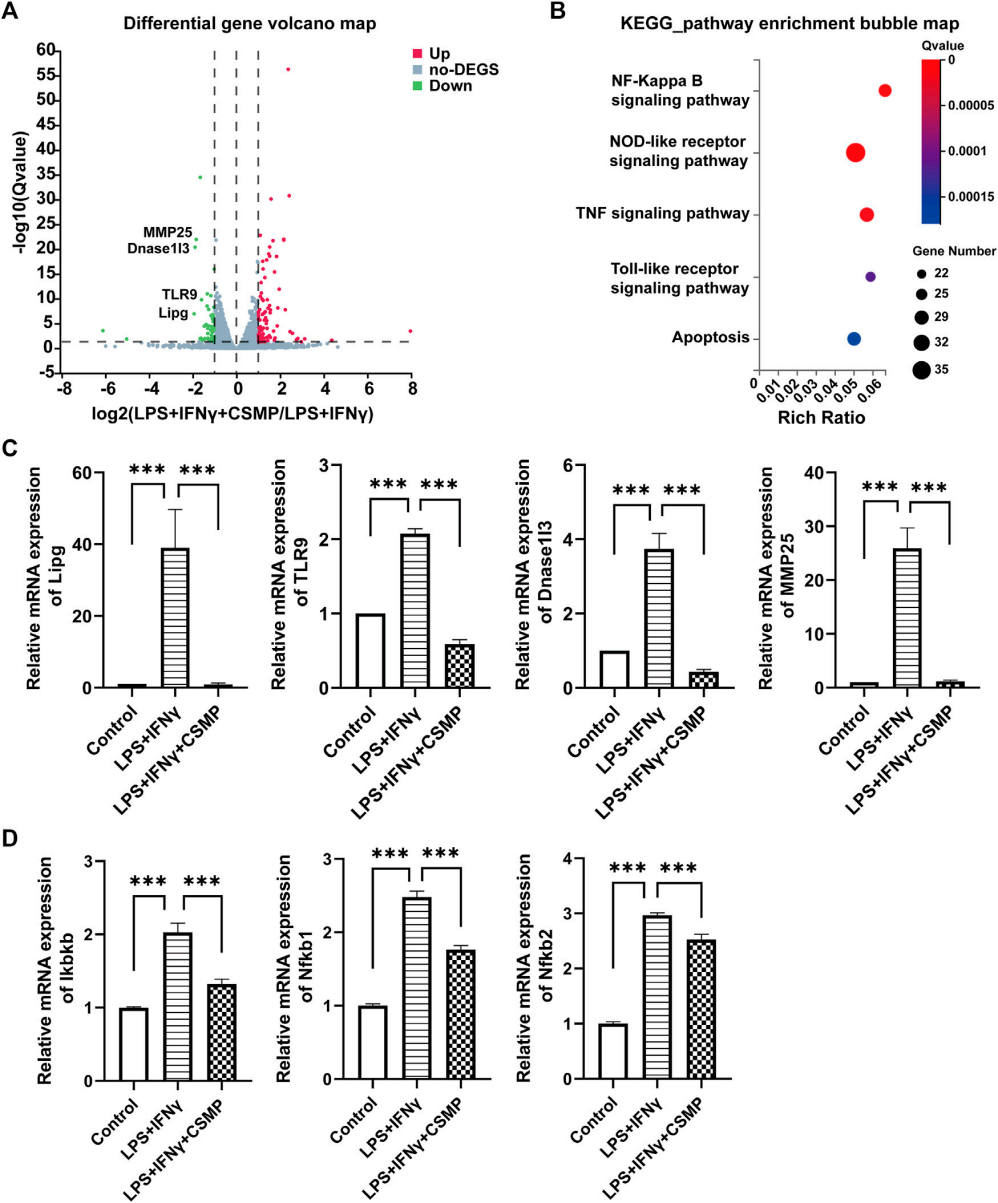
The mRNA sequencing and the RT-qPCR results for the pro-inflammatory factors stimulated BMMs after cocultured with CSMP hydrogel. **(A)** DEGs’ volcano plot, **(B)** Enriched items with KEGG analysis method. **(C)** RT-qPCR results for mRNA expression level for pro-inflammatory related genes, Lipg, TLR9, Dnase1I3, MMP25. **(D)** RT-qPCR results of mRNA expression level for Ikbkb, Nfkb1, Nfkb2 genes, and these genes are related to NF-κB signaling pathway. (*n* = 3, ***p < 0.001).

**FIGURE 6:**
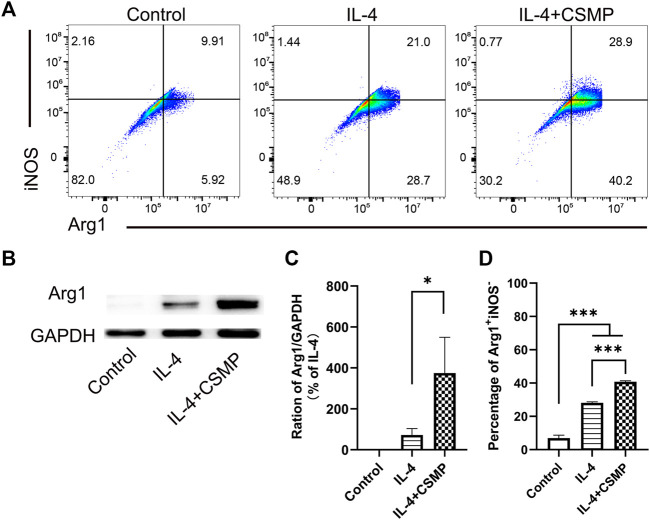
IL-4 treated BMMs’ polarization behavior when cocultured with CSMP hydrogel. **(A, D)** Flow cytometry and the statistical analysis results for the percentage of Arg1^+^iNOS^−^. **(B,C)** Western blot assay for the Arg1 proteins and the statistical analysis result for Arg1 protein expression. (*n* = 3, *p < 0.05, ***p < 0.001).

The pro-inflammatory factor-treated BMMs were co-cultured with hydrogels, and the expression of pro-inflammatory genes was investigated using RT-qPCR. The RT-qPCR results were presented in Figure 4. In the results, the pro-inflammatory genes expression, including IL-1β, IL-6, TNF-α, CCL2, IL-12β, iNOS, and CD86 were detected. As shown, all of these pro-inflammatory genes were upregulated after being treated with LPS and IFNγ, but when cultured with CSM or CSMP hydrogels, their expression was downregulated, particularly in the case of the CSMP hydrogel. This result demonstrated that the CSMP possessed a greater ability to inhibit the inflammatory response of LPS and IFNγ treated BMMs compared with the CSM hydrogel. Further, the mRNA sequencing assay for the BMMs was also performed to explore the potential signaling pathway for the CSMP hydrogel prohibiting the inflammatory response of LPS and IFNγ treated BMMs. The results were showed in Figure 5. From differentially expressed genes’ (DEGs’) volcano plot in Figure 5A, the pro-inflammatory genes, including Mmp25, Dnase1l3, TLR9 and Lipg, were downregulated for the CSMP + LPS + IFNγ group when compared with the LPS + IFNγ group. Figure 5C shows that the expression of the Lipg, TLR9, Dnase1l3, and Mmp25 genes was higher in CSMP hydrogel treated BMMs than in LPS and IFNγ treated BMMs. The KEGG-enriched signaling pathway result in Figure 5B displayed that the most promising signaling pathway for the CSMP hydrogel prohibiting the inflammatory response of LPS and IFNγ treated BMMs was the NF-κB signaling pathway. Furthermore, the RT-qPCR results in Figure 5D for characteristic gene expression in the NF-κB signaling pathway revealed that Ikbkb, Nfkb2, and Nfkb1 gene expression was lower in CSMP hydrogel treated cells than in LPS and IFNγ treated BMMs. The Western blot results in Supplementary Figure S2 showed that CSMP hydrogel-cultured LPS and IFNγ-treated BMMs had lower IL-1β protein expression than LPS and IFNγ-treated BMMs alone. Besides, the flow cytometry for the LPS and IFNγ treated BMMs cultured in CSMP hydrogel was investigated to identify the percentage of Arg1 vs. iNOS proteins, and the results were presented in Figure 6. The proportion of Arg1^+^iNOS^−^ for CSMP-treated BMMs was 40.2%, which was higher than 28.7% for IL-4-treated cells and 5.92% for the control group, according to cytometry results (Figures 6A, D), and the statistical analysis result in Figure 6D confirmed this result. The Western blot assay for Arg1 protein expression results in Figures 6B, C showed that the Arg1 protein expression for CSMP-cultured IL-4-treated BMMs was higher than that of IL-4 treated BMMs.

### 3.6 *In vivo* skin defect repair promotive property of CSMP hydrogel

**FIGURE 7:**
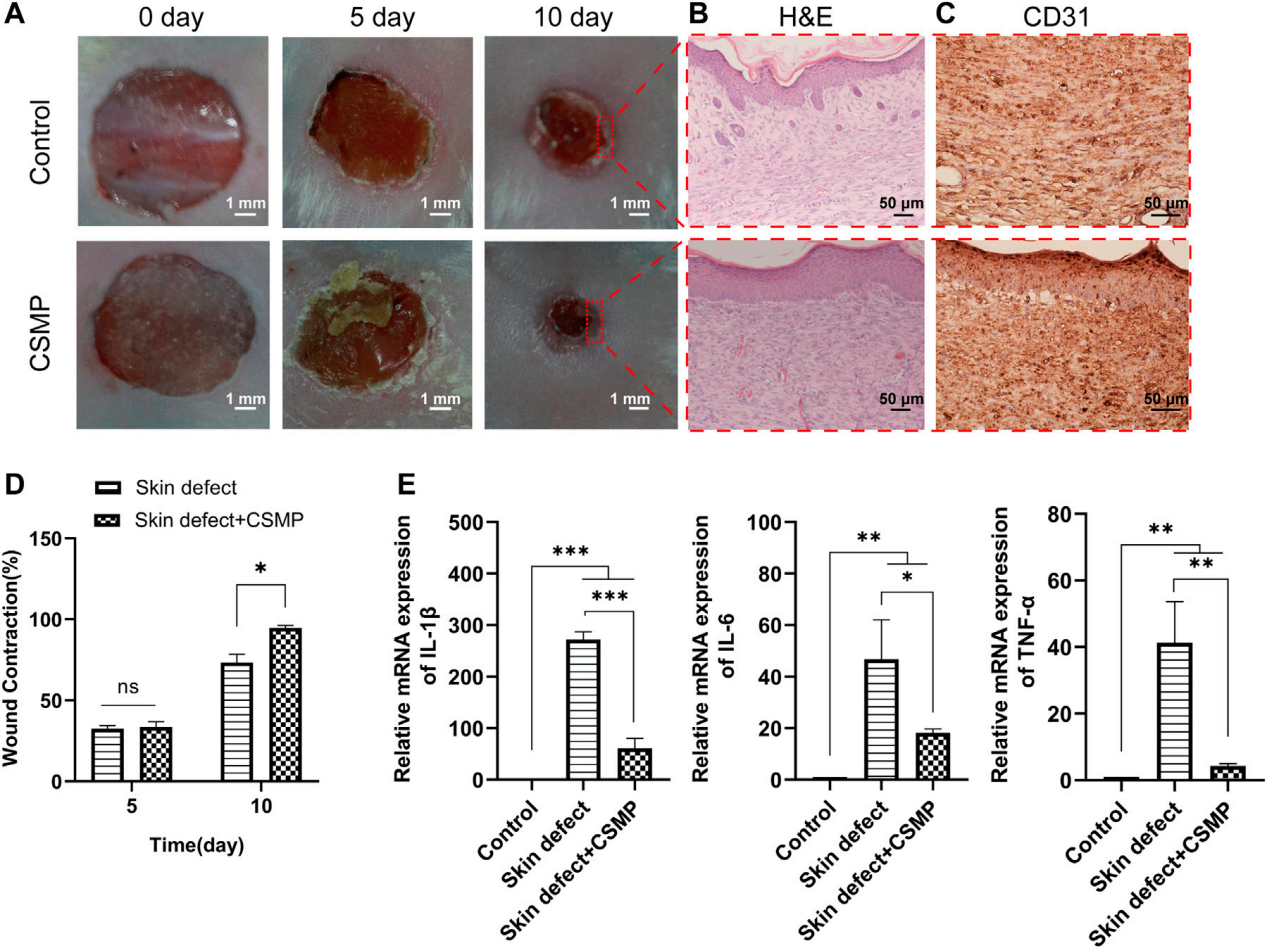
*In vivo* study for the CSMP hydrogel implanted in the 8 mm diameter wound defect area for 5 and 10 days. **(A, D)** Macroscopical images for the defect area and the calculated wound contraction after different implantation time points, **(B,C)** H&E and CD31 immunohistochemical staining for the wound defect area after 10 days implantation of CSMP hydrogel, **(E)** mRNA expression of inflammatory factor IL-1β, IL-6 and TNFα for the wound defect area after 10 days of implantation. (*n* = 4, ns: no significance, *p < 0.05, **p < 0.01, ***p < 0.001).

**FIGURE 8:**
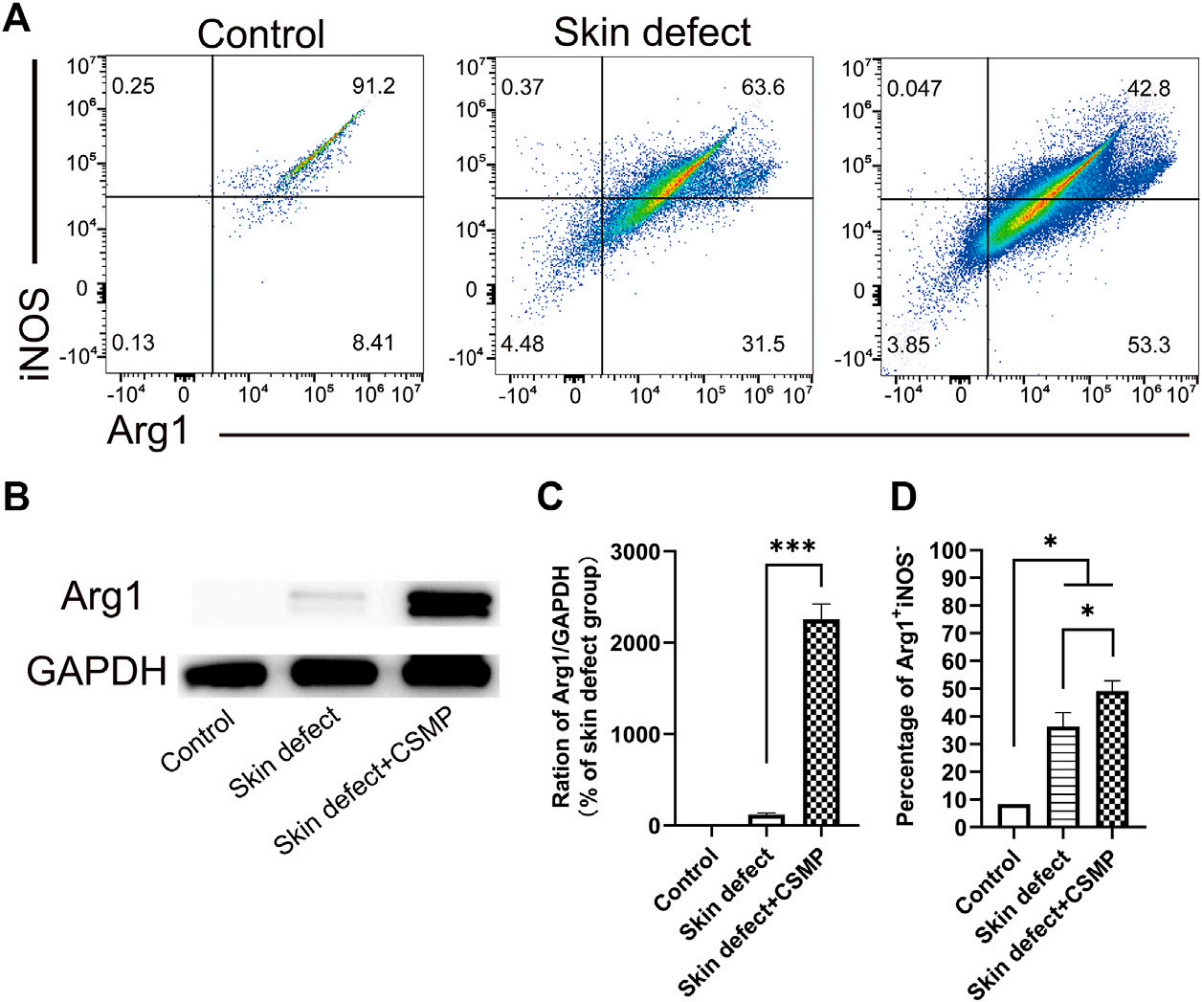
Flow cytometry and Western blot results for the wound defect tissue after 10 days implantation of CSMP hydrogel in comparison with no implanted defect area and no defect control groups. **(A)** and **(D)** Flow cytometry and statistical analysis results for the percentage of Arg1^+^iNOS^−^. **(B)** and **(C)** Western blot result for Arg1 protein expression and the statistical analysis. (*n* = 3, *p < 0.05, ***p < 0.001).

The *in vivo* skin defect repair promotive property of CSMP hydrogel was investigated, and the results were presented in Figures 7, 8. According to the macroscopical images in Figure 7A, the skin defect area gradually decreased over time, and the defect area for the CSMP hydrogel implanted was obviously smaller than that of the control group after 10 days of implantation; the statistical analysis result in Figure 7B confirmed this observation. The H&E staining result in Figure 7A showed that more lilac-like stained areas and more stained cells were present in the CSMP hydrogel implanted skin in comparison with control group; besides, the CSMP hydrogel implanted skin presented more smooth defect edge when compared with the control group. Figure 7A shows that the CSMP hydrogel group had more stained brown areas than the control group due to CD31 immunohistochemical staining. Further, the RT-qPCR result for the defect area after implanting with CSMP hydrogel was preformed, and the results were presented in Figure 7C. The mRNA expression level of IL-1β, IL-6, and TNF-α genes in the CSMP hydrogel implanted group was lower than that of the skin defect and blank control groups.

The flow cytometry results for the wound defect area after CSMP implanted for 5 days were also investigated, and the results were showed in Figure 8. The percentage of Arg1^+^iNOS^−^ in macrophages was higher in the CSMP implanted defect area than in the no implanted defect area and control groups. The screen process for macrophages in wound defect area was presented in Supplementary Figure S3. FSC-A, 7-ADD, APC-Cy7, Alexa Fluor 488, and PE-Cy7 markers were utilized to screen and identify the macrophage phenotype. Western blot results in Figures 8B, C showed Arg1 protein expression for the CSMP implanted defect area was higher than that of the sole defect area and the no defect control group.

## 4 Discussion

Wound defects were common in clinics, and some of these wounds would lead to a series of serious complications and even death (Pan et al., 2019). As a result, clinicians are always striving for rapid wound defect repair. Phosphocreatine has been confirmed to have the ability to modulate the macrophage phenotype, and in human skin, there are large quantities of phosphocreatine (Zemtsov, 2007), which plays an important role in protecting the skin from damage or other skin conditions. To our best knowledge, this study was the first attempt to utilize the synthesized phosphocreatine-grafted chitosan hydrogel to promote wound healing. The synthesis of phosphocreatine-functionalized methacryloyl chitosan CSMP and its hydrogel was gentle and straightforward.

The synthesized CSMP hydrogel presented the appealing physicochemical properties needed for wound defect healing. The lyophilized CSMP hydrogel possesses superior water uptake ability versus the lyophilized CSM hydrogel, which might be attributed to the larger pore size of the lyophilized CSMP hydrogel in comparison with the lyophilized CSM hydrogel (confirmed from surface SEM images). The large water uptake amount of this CSMP lyophilized hydrogel would be beneficial for the absorption of extraneous tissue fluid in the wound defect area, keeping the wound dry and therefore inhibiting bacteria breeding. During the *in vitro* immersion progress in PBS solution, the CSMP hydrogel showed higher compressive strength and modulus than the CSM hydrogel, which could be due to the phosphate groups in the grafted phosphocreatine combining with amino groups in chitosan via a hydrogel bond or other physical bond, enhancing the degree of crosslinking of the hydrogel. Interestingly, during the *in vitro* immersion process, the compress strength and modulus for these hydrogels increased after 7 days of immersion and then gradually decreased, which could be attributed to the ions in PBS physically combining with amino groups or phosphate groups in CSM or CSMP hydrogels, strengthening the compressive properties of the hydrogel; as immersion in PBS solution progressed, the hydrogels would gradually degrade.

Macrophages are widely distributed in different tissues and play an important role during the damaged tissue repair process. Typically, macrophages have two phenotypes: pro-inflammatory M1 and anti-inflammatory M2. The monocytes in the surrounding area would migrate to the damaged tissue and differentiate as pro-inflammatory M1 macrophages, which function as initial tissue defense and secrete pro-inflammatory factors, aggravating the inflammatory response and eventually inhibiting damaged tissue repair. As tissue repair progressed, the monocytes would differentiate as anti-inflammatory M2 macrophages, which would restore the tissue and inhibit damaged tissue repair. Therefore, inhibiting the inflammatory response of macrophages is essential to promote wound healing. The CSMP hydrogel inhibited the mRNA expression of pro-inflammatory factors (IL-6, TNF-α, and IL-1β), chemokines CCL2 and CD86 when co-cultured with LPS and IFNγ stimulated macrophages in this study, and the inhibitory effect of the CSMP hydrogel was better than that of the CSM hydrogel. TNF-α is a type II homotrimeric membrane protein (Yang et al., 2011; Adams et al., 2016) that plays a key regulatory role in inflammation and host defense against bacterial infection (Chen S. Y. et al., 2022), and IL-1β is a multifunctional cytokine involved in many immune and pro-inflammatory responses (Rastogi et al., 2018). The CCL2/CCR2 axis is closely associated with high levels of immune activation and inflammation (Yang et al., 2011). CD86, a member of the B7 cell surface ligand family, is expressed in resting monocytes, dendritic cells, and activated B lymphocytes, which is further upregulated in the presence of inflammation (Adams et al., 2016). These results suggest that CSMP has a powerful anti-inflammatory effect. Further, the mRNA sequencing results confirmed that the expressions of Mmp25, Lipg, TLR9, and Dnase1l3 were significantly downregulated in the CSMP + LPS + IFNγ group compared with the LPS + IFNγ group. These genes are closely associated with pro-inflammatory responses. MMP25 from Japanese sea bass (Lateolabrax japonicus) was involved in the pro-inflammatory response (Chen S. Y. et al., 2022). Lipg is an esterase with enormous immunogenic properties that elicits strong humoral responses as well as cell-mediated immunity (Rastogi et al., 2018). TLR9 can undergo proteolytic cleavage in endosomes after ligation by CpG DNA. Cleaved TLR9 then recruits MyD88 to activate NF-κB and MAPKs and induce the production of proinflammatory cytokines, or to induce the production of type I IFN by activating IRF7 (Kawai and Akira, 2010; Pandey et al., 2014). DNase1l3 is secreted by innate immune cells and may play a critical role in maintaining tissue homeostasis and preventing the development of autoimmunity (Inokuchi et al., 2020). CSMP may exert anti-inflammatory effects through these genes. Besides, CSMP hydrogel inhibited the secretion of pro-inflammatory factors by macrophages mainly through the TNF signaling pathway, the chemokine signaling pathway, the NOD-like receptor signaling pathway, and the NF-κB signaling pathway. These signaling pathways play an important role in the occurrence and development of inflammation. CSMP can significantly inhibit the expression of TNF signaling pathway-related genes such as Casp3, Socs3, Cxcl10, IL15, Ccl12, Tnfaip3, and Tnfrsf1a. TNF signaling pathway inhibition is beneficial in preventing tissue damage caused by inflammation and promoting tissue repair (Luo et al., 2020). Chemokines play an important role in the activation of monocytes and lymphocytes at sites of inflammation. In addition, CSMP can significantly inhibit NF-κB signaling pathway related genes such as IKbkb, Myd88, Nfkb1, Nfkb2, Vcam1, Cd40, Traf1, Icam1 and Cflar. The NF-κB signaling pathway is a classical signaling pathway that regulates inflammation and immunity (Wang et al., 2019). When macrophages are infected with LPS, pathogenic microorganisms, and so on, they become activated, resulting in the phosphorylation of the transcription product NF-κB and nuclear translocation, as well as the production of inflammation-related target genes such as IL-6 and TNF-α (Gupta et al., 2010; Hoesel and Schmid, 2013).

The animal study confirmed that CSMP could promote rapid wound healing and inhibit the expression of pro-inflammatory factors (IL-1β, IL-6, and TNF-α). CSMP can also promote the high expression of Arg1 in the macrophages of damaged skin tissue. Arg1 is a biomarker of M2 macrophages (Murray, 2017). Previous studies have shown that M2 macrophages play an important role in the remodeling stage of tissues (Xie et al., 2019). Bioactive materials inhibit the secretion of pro-inflammatory factors by M1 macrophages, and at the same time promote the polarization of macrophages to the M2 type, which can promote tissue repair. This indicates that the effect of CSMP in promoting skin repair may be produced by inhibiting the secretion of pro-inflammatory factors in BMMs and promoting the polarization of macrophages to the M2 type. Taken together, this phosphocreatine-grafted chitosan hydrogel showed anti-inflammatory property, mechanical stability, and lyophilized hydrogel possessed the high water absorption, which are beneficial for the wound healing. The limitations of study should be mentioned here and the further study will concentrate on these aspects. First, the *in vitro* and *in vivo* biodegradation behavior should be investigated. Second, the anti-bacterial and angiogenic properties for the wound dressing are also quite important, and the further study will include these investigations with/without inclusion of bioactive components in this hydrogel system. Last but not the least, the refractory wounds are common in clinic and the animal mode in further study will focus on this.

## 5 Conclusion

The synthesized CSMP hydrogels (in lyophilized state) exhibited a porous structure, and the pore size was ca. 200 μm. The lyophilized CSMP hydrogel possessed a higher water uptake versus the CSM control hydrogel. During the *in vitro* immersion progress, the compressive strength and modulus of the CSMP hydrogel were higher than those of the CSM control hydrogel, and with immersion progress, the compressive strength and modulus of these hydrogels were increased after 7 days of immersion and then gradually decreased. The cell culture study revealed that the CSMP hydrogel can inhibit the mRNA expression of pro-inflammatory factors IL-1β, IL-6, and TNF-α, inflammatory chemokines CCL2, and pro-inflammatory-related genes Lipg, MMP25, TLR9, and Dnase1L3, and that the CSMP hydrogel’s anti-inflammatory property may be mediated by the NF-κB signaling pathway. Further, the CSMP hydrogel also promoted mouse wound healing. Taken together, the CSMP hydrogel showed great potential for wound defect healing in clinics.

## Data Availability

The original contributions presented in the study are included in the article/Supplementary Material, further inquiries can be directed to the corresponding authors.
