# Supplementary material for: Advanced phosphocreatine-grafted chitosan hydrogel promote wound healing by macrophage modulation
Source: Front Bioeng Biotechnol. 2023 May 12;11:1199939. doi: 10.3389/fbioe.2023.1199939 (PMC10213409; doi:10.3389/fbioe.2023.1199939)
Supplement: Supplementary file 1 [file DataSheet1.docx]

Supplementary Material

# Supplementary Figures


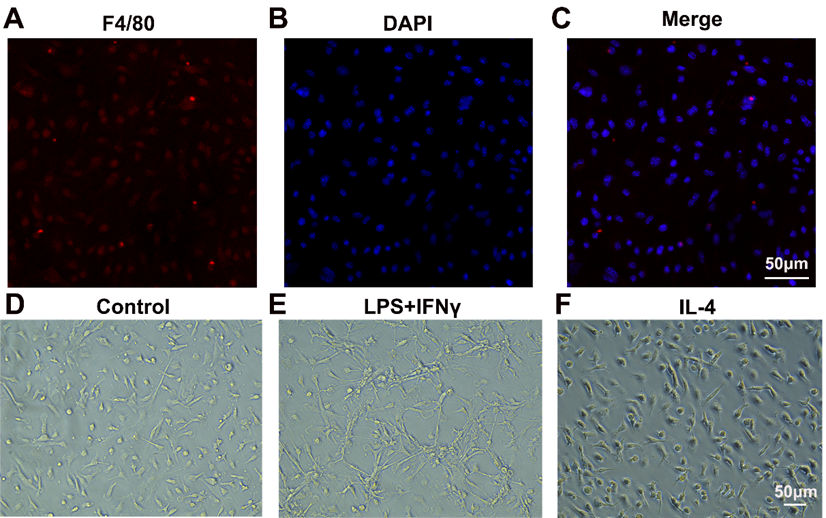


**Figure S1:** Identification for the extracted BMMs. (A)-(C) Fluorescent image for F4/80 stained BMMs, F4/80 is the surface marker for BMMs. (D)-(F) Cell morphology of extract cells, and LPS+IFNγ-treated BMM or IL-4 treated BMMs for 24 hours.


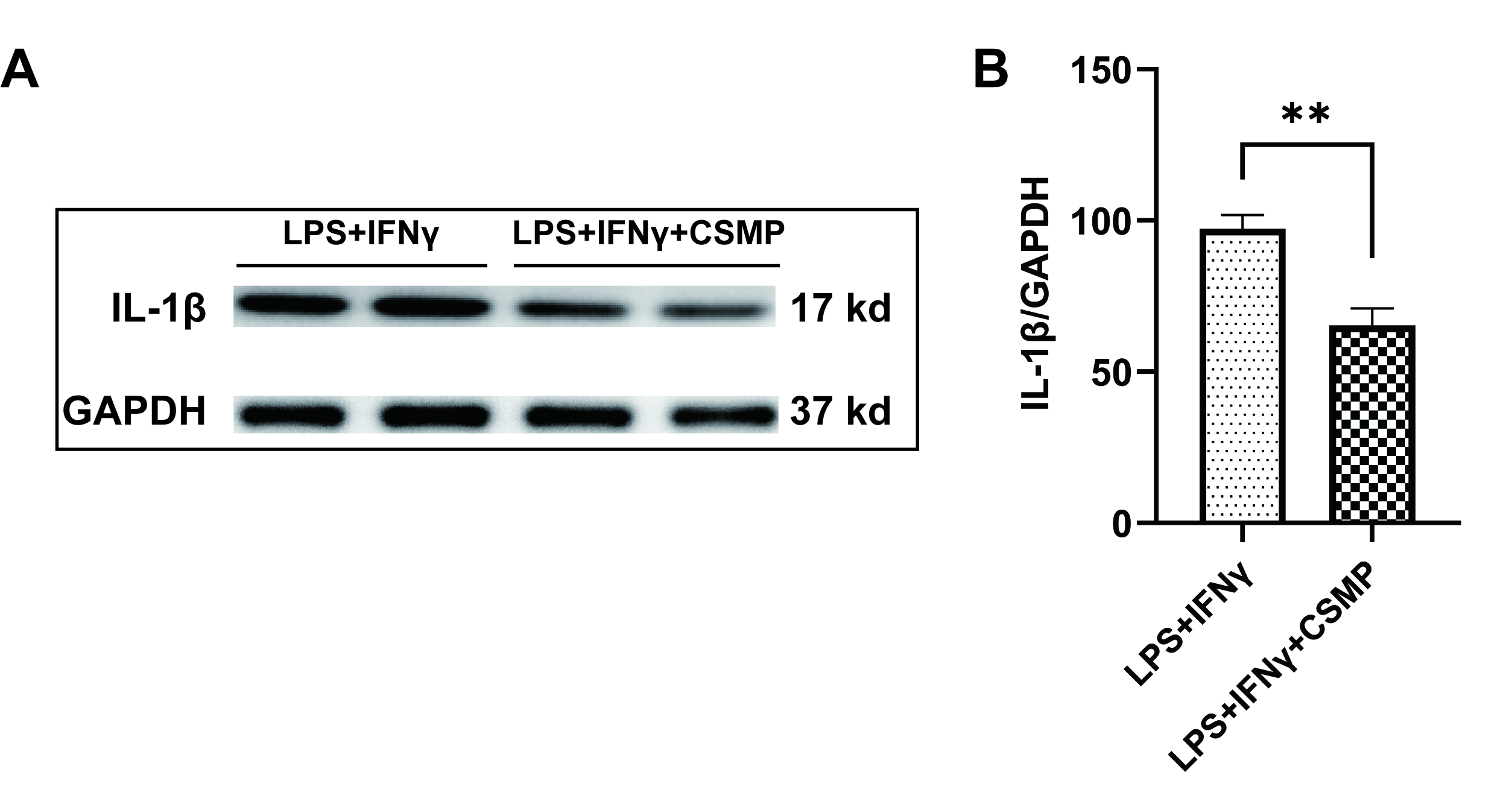


**Figure S2:** Western blot assay of IL-1β protein for the LPS+IFNγ-treated BMMs’ after cocultured with CSMP hydrogel.


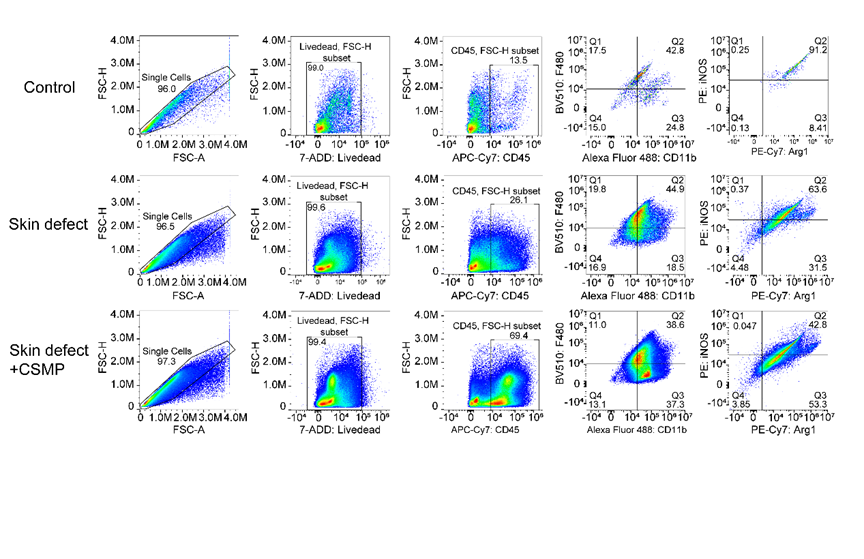


**Figure S3:** Flow cytometry of macrophages screening process in wound defect tissue after hydrogels implanted for 5 days.
